# Adjacent-level biomechanics after single-level anterior cervical interbody fusion with anchored zero-profile spacer versus cage-plate construct: a finite element study

**DOI:** 10.1186/s12893-020-00729-4

**Published:** 2020-04-06

**Authors:** Xin-Feng Li, Lin-Yu Jin, Chao-Ge Liang, Hong-Ling Yin, Xiao-Xing Song

**Affiliations:** 1 Department of Orthopaedic Surgery, Baoshan Branch of Renji Hospital, School of Medicine, Shanghai Jiaotong University, No. 1058, Huan Zheng Bei Rd, Shanghai, 200444 P.R. China; 2 Department of Spinal Surgery, Peking University People’s Hospital, Peking University, Beijing, 100044 China; 3 Department of Orthopaedic Surgery, Shanghai Xijiao Orthopaedic Hospital, Shanghai, 200336 China; 4 School of Materials Science and Engineering, Shanghai Jiaotong University, No. 1954, Huashan Rd, Shanghai, 20030 P.R. China; 5 Department of Anesthesiology, Ruijin Hospital, Shanghai Jiaotong University School of Medicine, 197 Ruijin Er Lu, Shanghai, 200025 China

**Keywords:** Cervical spine, Adjacent segment degeneration, Anterior cervical discectomy and fusion, Plate profile, Finite element analysis

## Abstract

**Background:**

The development of adjacent segment degeneration (ASD) following ACDF is well established. There is no analytical study related to effects of plate profile on the biomechanics of the adjacent-level after ACDF. This study aimed to test the effects of plate profile on the adjacent-level biomechanics after single-level anterior cervical discectomy and fusion (ACDF).

**Methods:**

A three-dimensional finite element model (FEM) of an intact C2–T1 segment was built and validated. From this intact model, two instrumentation models were constructed with the anchored zero-profile spacer or the standard plate-interbody spacer after a C5-C6 corpectomy and fusion. Motion patterns, the stresses in the disc, the endplate, and the facet joint at the levels cephalad and caudal to the fusion were assessed.

**Results:**

Compared with the normal condition, the biomechanical responses in the adjacent levels were increased after fusion. Relative to the intact model, the average increase of range of motion (ROM) and stresses in the endplate, the disc, and the facet of the zero-profile spacer fusion model were slightly lower than that of the standard plate-interbody spacer fusion model. The kinematics ROM and stress variations above fusion segment were larger than that below. The biomechanical features of the adjacent segment after fusion were most affected during extension.

**Conclusions:**

The FE analysis indicated that plate profile may have an impact on the biomechanics of the adjacent-level after a single-level ACDF. The impact may be long-term and cumulative. The current findings may help explain the decreasing incidence of ASD complications in the patients using zero-profile spacer compared with the patients using cage and plate construct.

## Background

Anterior cervical decompression and fusion (ACDF) is a standard procedure, which can achieve good to excellent clinical results in the treatment of cervical radiculopathy and myelopathy [1–5]. Anterior stabilization techniques have been described since 1970 [6], however, the development of adjacent segment degeneration (ASD) following ACDF is well established, and ASD approximately occurs in 25% of patients within the first 10 years following fusion [7–13]

Currently, either bone or cage spacer with anterior plate and screw stabilization is the standard fusion procedure during ACDF. Although the pathogenesis of this subsequent ASD remains unknown, the development of ASD might be multifactorial. ASD changes may be related to the biomechanical effects of cervical fusion and/or the biology of cervical degeneration [11, 14–16]. After fusion, the altered biomechanics at the adjacent levels could lead to increased mobility [17–20], increased loading [21], or increased intradiscal pressure [9, 22], and, ultimately, accelerated disc degeneration [23–25].

Cage and plate construct or stand-alone cage is a popular procedure used in ACDF procedure. Recent clinical observation showed a higher incidence of ASD in ACDF with plate and cage construct (PCC) than that in stand-alone cage [26]. Clinical risk-Factor analysis of ASD following ACDF indicated that plate-to-disc distance might be an independent predisposing factor for the occurrence of ASD [27–31]. However, there is no good analytical study related to the effect of plate profile on the biomechanics of the adjacent-level after ACDF. An anchored zero-profile spacer (Zero-P) has been developed to reduce the potential risk of complications after anterior cervical fusion with plating. To clarify the effect of plate profile on the biomechanics of the adjacent-level after ACDF, the present study aimed to observe the different biomechanical features of the adjacent segment after fusion with the Zero-P and the PCC.

## Methods

### Study design

In this study, a computational biomechanical analysis was used to describe and predict the effect of plate profile on the mechanical behavior of the adjacent segment after ACDF.

### Development of finite element model (FEM)

A 3-dimensional FEM of a normal C2-T1 segment was created in this study. A 38-year-old male patient was selected in our simulation. This study was performed in accordance with the ethical standards of the Institutional Ethics Committee of our hospital. Written informed consent was obtained from the patient. The model was developed from the computed tomographic (CT) scan of this male subject. Model creating methods were used as we previously described [32–35]. Lordosis of C2-C7 in current model was 23.7°. The measured Cobb angle was in the reported range of the subaxial cervical spine [36]. The global xyz coordinate system was set with the positive z-axis acting along the rostral-caudal extent of the spine pointing up, the positive x-axis was perpendicular to the z-axis pointing right, and the positive y-axis was perpendicular to the x-axis and z-axis pointing backward. The model components included cortical bone, cancellous bone, bony posterior elements, annulus fibrosus (AF), nucleus pulposus (NP), posterior facets, end plates, anterior longitudinal ligament, posterior longitudinal ligament, ligamentum flavum, interspinous ligament, and capsular ligaments. The material properties were assumed to be homogeneous and isotropic according to the published literature [37–42]. The annular fibers embedded in the ground substance were assembled in a crisscross manner. The facet joint was created as a nonlinear three-dimensional contact problem using surface-to-surface contact elements. Surface to surface contact algorithm is used in defining facet joint interaction and friction coefficient was assumed to be 0.1 [43]. The initial material properties were based on previous studies as shown in Table 1.

**Table 1:** Material properties of the spinal structures and instrumentations

Description	Element Type	Young’s Modulus (MPa) E	Poisson Ratio μ	Cross Sectional Area (mm^2^)
Cortical bone	Shell elements	12,000	0.29	–
Cancellous bone	3-D solid elements (4 node)	450	0.25	–
Facet cartilage	3-D solid elements (4 node)	10.4	0.4	–
Annulus fibers	3-D solid elements (8 node)	48	0.4	–
Nucleus pulposus	3-D solid elements (8 node)	1.0	0.49	–
Endplate	3-D solid elements	500	0.4	–
Anterior longitudinal ligament	3-D tension truss elements	30	0.3	33
Posterior longitudinal ligament	3-D tension truss elements	20	0.3	33
Interspinous ligament	3-D tension truss elements	1.5	0.39	13
Ligamentum flavum	3-D tension truss elements	5	0.3	50.1
Capsular ligaments	3-D tension truss elements	20	0.3	46.6
PEEK cage	3-D solid elements (4 node)	3600	0.3	–
Titanium plate	3-D solid elements (4 node)	110,000	0.3	–
Screw	3-D solid elements (4 node)	110,000	0.3	–

### Model validation

Range of motion (ROM) of each cervical segment was the major indicator for FEM validation. Subaxial ROM and functional spinal unit ROM were calculated. The subaxial ROM was defined as the measurement of the total motion between the C2 and C7 vertebrae. The functional spinal unit ROM, intersegmental motion, was the motion between two adjacent vertebrae. The predicted ROM (movement in sagittal plane, coronal plane and axial plane) were compared with the in vivo and in vitro data obtained from healthy normal cervical spine [44–47]. All simulations had the same boundary and loading conditions with the controlled experimental studies. Boundary conditions, defined as the inferior surface of T1 vertebra was fully constrained in all loading conditions. To simulate the upper head weight, a compressive preload of 100 N was applied to simulate physiologic compressive loads [42, 44]. The compressive preload was applied using the follower load technique [48]. After applying the preload, a 2-Nm bending moment was applied to the superior surface of C2. Simulations were conducted using Abaqus (Simulia, Providence, RI). Subaxial ROM and functional spinal unit ROM were recorded.

### Fusion models simulation and biomechanical changes

Fusion models were developed from the intact cervical spine model. Discectomy and accommodate the implant-bone graft assembly were simulated. To simulate single-level ACDF, the anterior longitudinal ligament, the C5-C6 intervertebral disc and cartilaginous endplate were removed, keeping posterior elements, associated ligaments and facet surfaces, and uncovertebral joints intact. Three conditions were modeled: intact model, 4-screw anchored Zero-P fusion model (ZeroP, DePuy Synthes Spine, Raynham, Massachusetts), and the standard PCC fusion model. The screw-plate and screw-bone interface were completely fixed in all directions. In the standard PCC, a rigid anterior cervical plate was used, plate (width 16 mm, thickness 2 mm) and fixed screws (diameter 4 mm, length 14 mm) were rigidly fixed in the operative segment. To maintain the sagittal alignment after surgery, the same PEEK interbody spacer (width 15 mm, length 16 mm, and height 6 mm) was used. Cervical spine alignment was assessed with the C2–7 Cobb angle. The angle was 22° for both fusion models. To simulate a standard surgical procedure, we placed the plate at least 5 mm away from the adjacent disc spaces. The above and below distance of the plate to the adjacent disc were 5.5 mm and 5 mm in PCC model, whereas, 13.5 mm and 12.5 mm in anchored Zero-P model. In the Zero-P system, fixed screws (diameter 3 mm, length 16 mm) were adopted. The titanium alloy plate and PEEK material properties were assigned to the respective implants (Table 1). Biomechanical segmental response in different model conditions was simulated. A torque of 2 Nm was applied with a 100-N axial preload. The inferior surface of T1 was fully constrained along three perpendicular planes in all loading conditions. The biomechanical responses of adjacent segments at the cephalad and caudal levels of the operation level were assessed in terms of ROM, stresses in the endplate, disc and facet. ROM in sagittal plane, coronal plane and axial plane was measured. Stresses were calculated using the average von-Mises stresses. The facet stresses at a motion segment was defined as the average von-Mises stresses on the right and left articulating facets.

## Results

### Model validation

**Fig. 1:**
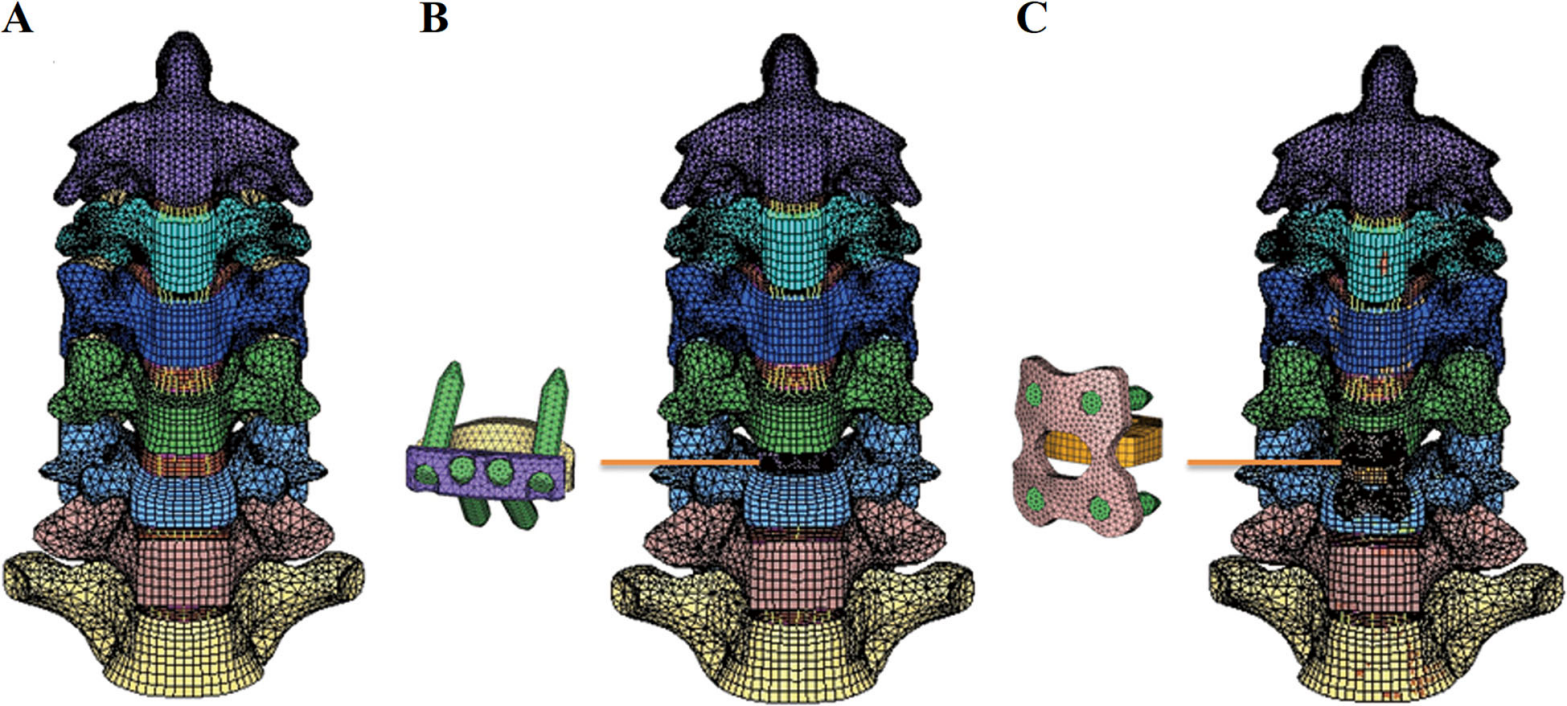
A three-dimensional finite element model of a healthy C2–T1 segment (**a**) and two fusion models with 4-screw Zero-P (**b**) and the standard PCC (**c**) at the C5–C6 segment

**Fig. 2:**
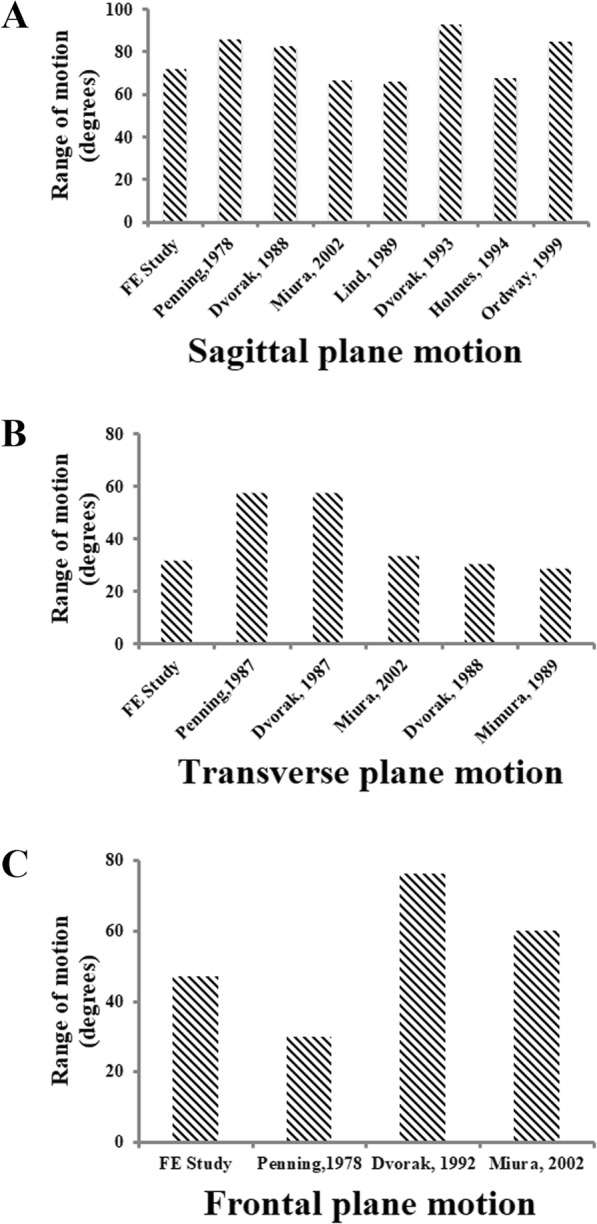
Comparison of the total subaxial ROM of FEM with the in vivo and in vitro studies in the sagittal plane (**a**), the transverse plane (**b**), and the frontal plane (**c**)

**Fig. 3:**
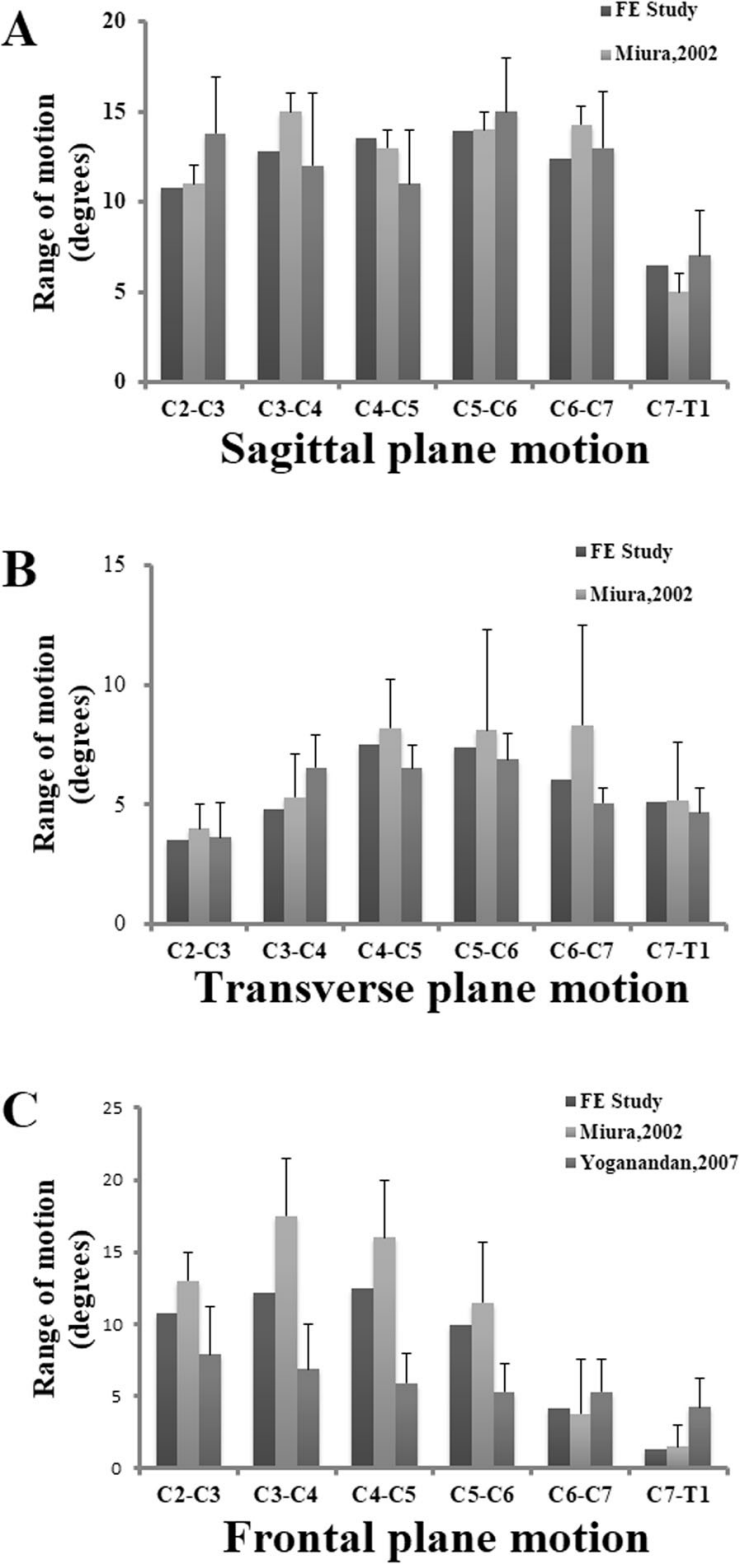
Comparison of the kinematic response of each segment of FEM with the in vivo and in vitro studies in the sagittal plane (**a**), the transverse plane (**b**), and the frontal plane (**c**)

The intact cervical spine FEM consisted of 152,608 elements and 41,797 nodes (Fig. 1a). The validation studies of the current FEM were shown in Figs. 2 and 3. Comparison of the total subaxial ROM was displayed in Fig. 2. The kinematic response of each segment of the FEM was compared with the corresponding in vivo and in vitro data [44–47] (Fig. 3). The total subaxial ROM of the normal FEM in the sagittal, the transverse, and the frontal planes were 72°, 32°, and 47° respectively. The segmental motions predicted by the normal C2–T1 FEM in the sagittal plane, transverse plane, and the frontal plane respectively were as follows: C2–C3 (10.8°, 3.5°, 10.8°,), C3–C4 (12.8°, 4.8°, 12.2°,), C4–C5 (13.5°, 7.5°, 12.5°,), C5–C6 (14.0°, 7.4°, 10.0°,), C6–C7 (12.4°, 7.1°, 4.1°,), and C7–T1 (6.5°, 5.6°, 1.3°,). The motions obtained from the present FEM were within standard deviation of the in vitro or in vivo study. The differences in segmental motions between the intact FEM and in vitro or in vivo data were small. Following validation, the C2–T1 FEM was then used to understand the biomechanical effects of plate profile on the biomechanical changes in adjacent segments (Fig. 1).

### Kinematics changes in the adjacent segments after fusion

**Fig. 4:**
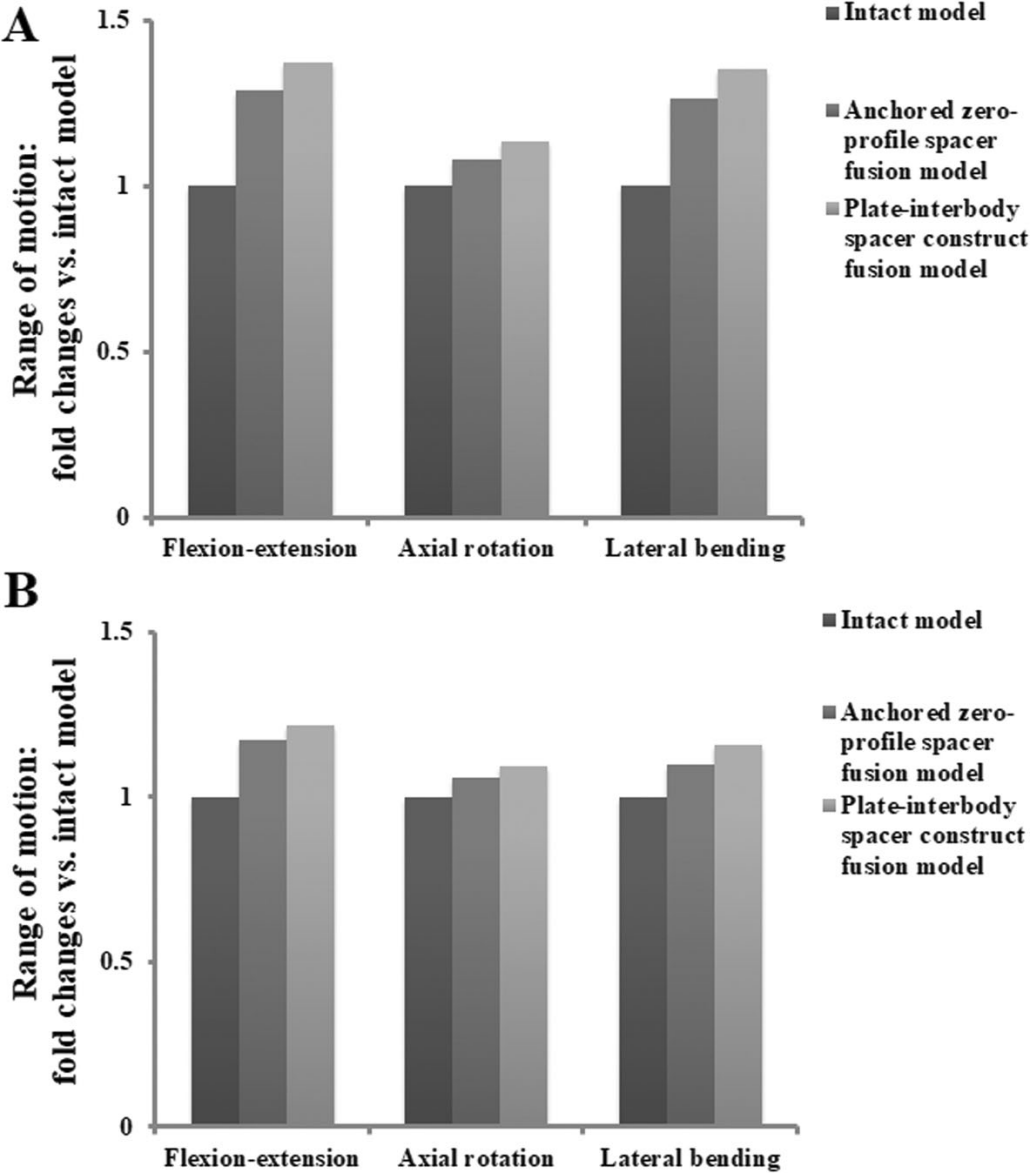
Kinematics changes relative to intact condition in the adjacent segment ROM, both at the proximal (**a**) and distal (**b**) adjacent levels

Kinematics changes relative to intact condition in the adjacent segment ROM, both at the proximal (Fig. 4a) and distal (Fig. 4b) adjacent levels were demonstrated. The ROM in both adjacent segments was greater after fusion than that in intact model. The highest increase was observed in the standard PCC fusion model, followed by the Zero-P fusion model. Compared with the intact model, the motions in the instrumentation models increased at segments adjacent to the fusion construct (C4–C5, C6–C7): the Zero-P fusion model (flexion-extension [29, 17%], axial rotation [8, 6%] and lateral bending [26, 10%]); and the PCC fusion model (flexion-extension [37, 22%], axial rotation [13, 9%] and lateral bending [35, 16%]). Motion changes were higher at the proximal adjacent levels than at the distal adjacent levels in the sagittal plane, the axial plane and the frontal plane. Both in the Zero-P fusion model and in the PCC fusion model, the motion of superior and inferior adjacent levels was most affected during movement in the sagittal plane.

### Intradiscal stress changes in the adjacent level

**Fig. 5:**
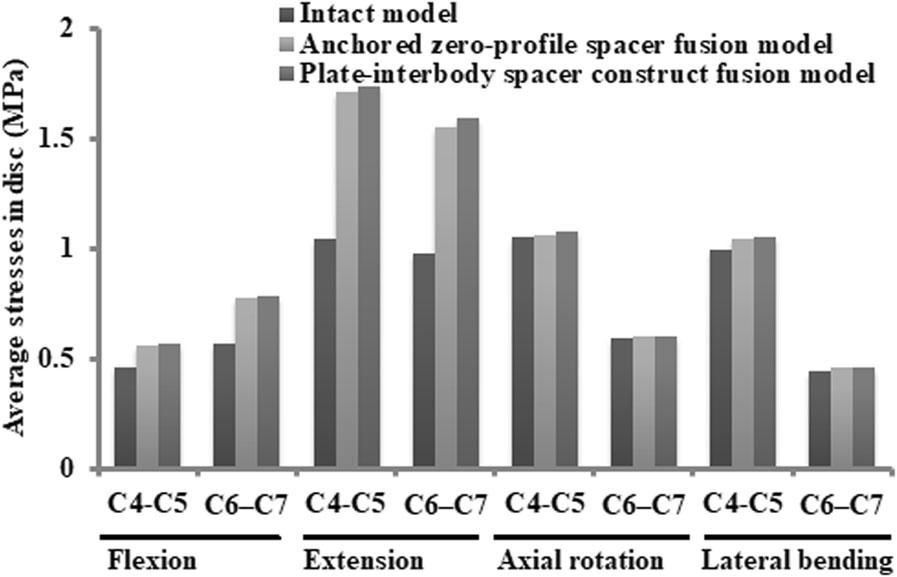
Stresses inside the superior C4–C5 and inferior C6–C7 discs of the intact model, the Zero-P fusion model, and the standard PCC fusion model

**Fig. 6:**
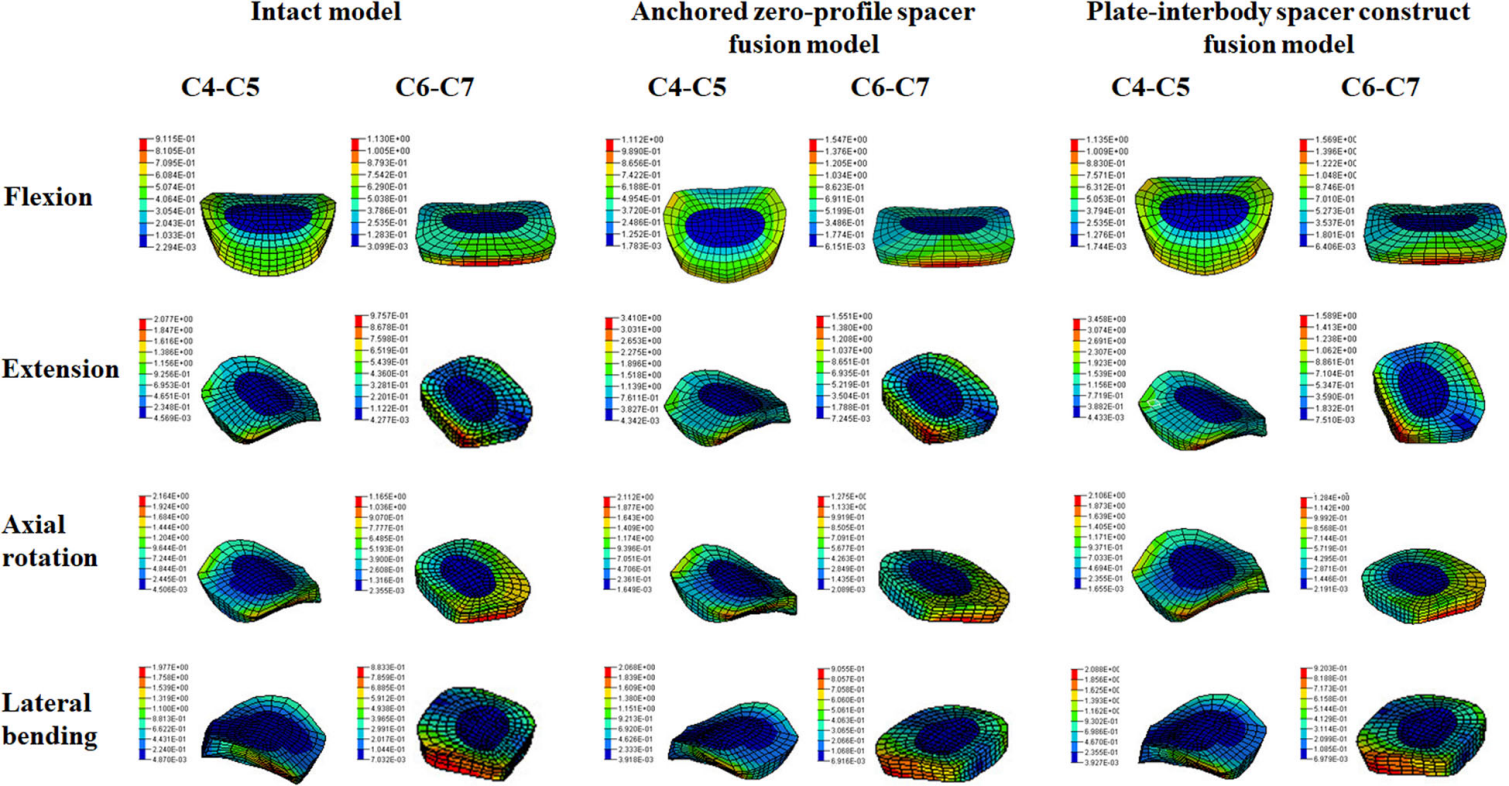
Intradiscal stress distribution features of C4-C5 and C6-C7 segments in the intact model, Zero-P model and PCC model in flexion, extension, axial rotation and lateral bending conditions. The colour changes from red to deep blue represent the stress variation from large to small on the stress nephogram

As shown in Fig. 5, compared with the intact model, the average disc stresses at the segments adjacent to the fusion construct (C4–C5, C6–C7) increased both in two instrumentation models. The disc stress increase in the fusion models was generally greater in the superior C4-C5 disc than that in the inferior C6-C7 disc during extension, axial rotation and lateral bending movement. In the C4-C5 disc, relative to the intact model, the average disc stress in the Zero-P and the PCC fusion model, increased by 21 and 24% during flexion; 64 and 66% during extension; 1 and 3% during axial rotation; and 5 and 6% during lateral bending. In the C6-C7 disc, when compared with the intact model, the average disc stress for Zero-P and PCC fusion model was 37 and 39% larger in flexion; 59 and 63% larger in extension; 2 and 2% larger in axial rotation; and 2 and 4% larger in lateral bending (Fig. 5b). Stresses of the superior C4-C5 and inferior C6-C7 discs were most affected in extension (Fig. 5). Intradiscal stress distribution features of C4-C5 and C6-C7 segments in the intact model, Zero-P model and PCC model under flexion, extension, axial rotation and lateral bending conditions were presented in Fig. 6. The maximum von Mises stress occurred at the anterior part of AF in flexion, at the posterolateral part in extension and at the lateral part in axial rotation and lateral bending movement.

### End-plate stress changes in the adjacent segments

**Fig. 7:**
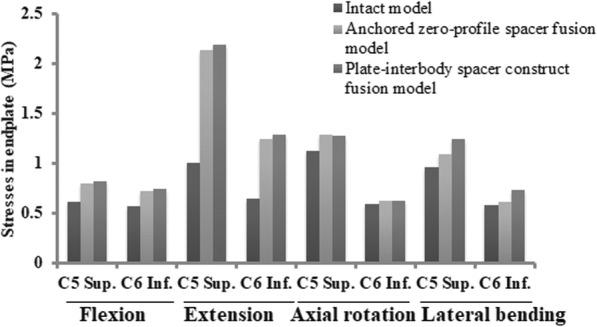
Stresses in the C5 superior and C6 inferior endplates of the intact model, the Zero-P fusion model, and the standard PCC fusion model

The C5 superior and C6 inferior end-plate stresses of two fusion models were higher than those of the intact model as shown in Fig. 7. In the fusion models, the end-plate stresses were highest during extension. The differences of the end-plate stresses between two fusion models were lowest during rotation. The increased stress values of C5 inferior end-plate relative to the intact model in the Zero-P fusion model were lower than that in the PCC fusion model in flexion (28, 33%), extension (113, 119%), and lateral bending (13, 30%). The increased stress values of C6 inferior endplate relative to the intact model in the Zero-P fusion model were also lower than that in the PCC fusion model in flexion (27, 32%), extension (90, 98%), and lateral bending (7, 28%).

### Facet load changes in the adjacent level

**Fig. 8:**
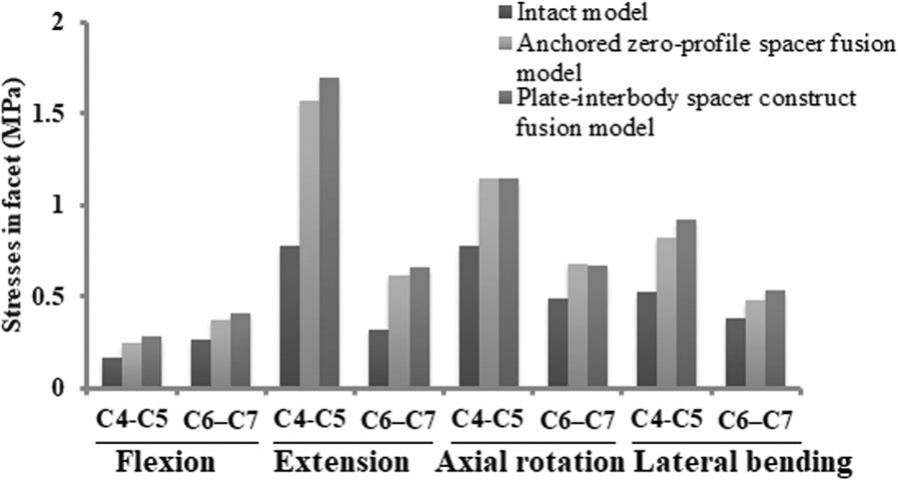
Stresses in the facets at the superior C4–C5 and inferior C6–C7 segments of the intact model, the Zero-P fusion model, and the standard PCC fusion model

The stress in the facet was found to be the greatest in two fusion models (Fig. 8). Compared with the intact model, the facet stress in the instrumentation models increased at the segments adjacent to the fusion construct (C4–C5, C6–C7): the zero-profile spacer fusion model (flexion [49, 40%], extension [102, 97%], axial rotation [48, 38%], and lateral bending [57, 26%]); the PCC fusion model (flexion [71, 52%], extension [117, 111%], axial rotation [48, 36%], and lateral bending [77, 39%]). The increased stress in the facet relative to the intact model was higher in the superior C4–C5 segment than in the inferior C6–C7 segment during flexion, extension, and lateral bending. The stresses were largest during extension.

## Discussion

Spinal arthrodesis biomechanically transfers additional stress to the adjacent segments, which may play a role in the development of ASD. The results obtained in the current computational investigation indicated that plate profile has an impact on the biomechanics of the adjacent-level after a single-level ACDF. The biomechanical responses in the endplate, the disc, and the facets in the adjacent levels were increased after fusion, compared with that in the intact model. Relative to the intact model, the average increase of ROM and stresses in the Zero-P fusion model were slightly lower than that in the standard PCC fusion model during flexion, extension, and lateral bending. The kinematics ROM and stress variations above fusion segment were larger than that below. The biomechanical features of the adjacent segment after fusion were most affected during extension.

Patients with spinal fusion are at greater risk for ASD and some of them are require additional surgery. ACDF with plating has become the most popular surgical approach for treatment of cervical spine disorders because anterior plates provide many advantages than without plating. As indicated in the present finite element study, ACDF impairs normal cervical biomechanics, alters mechanics at adjacent segments, resulting in higher stress and hypermobility in adjacent segments. Our results are in line with the reported data of the biomechanical studies on the behavior of the adjacent segments in the setting of different operation procedures [49–53]. Both the present and all of the reported results suggested that ASD might be related to iatrogenic biomechanics. Although the pathophysiology of ASD remains controversial, ASD is likely a multifactorial process that is not only driven by natural history, but also affected by the increased adjacent segment mobility and by the changes of mechanical environment [54–56]. It is well known that mechanical loads could induce intervertebral disc degeneration by initiating degeneration or by regulating cell-mediated remodeling events that occur in response to the mechanical stimuli [57]. Mechanical stimuli have an impact upon the cellular response to loading and upon changes that occur with aging and degeneration of the intervertebral disc [58, 59].

Low profile, integrated interbody cages design anchors to the vertebral bodies from within the disc space. In vitro biomechanical study showed that the anchored cage afforded biomechanical stability comparable to that of the standard plate-interbody cage [60, 61]. This design has greater advantages in decreasing incidence of postoperative dysphagia and ASD complications. As demonstrated in several clinical studies, the Zero-P group had a significantly reduced incidence of ASD compared with the PCC group [61–67]. Our kinematics simulation study showed a lower hypermobility in adjacent segments in the Zero-P fusion model than that in the standard PCC fusion model (Fig. 4). Excessive motion of a given motion segment unit has been postulated to lead to an increased risk of disc degeneration, due to mechanical forces exerted on adjacent segments from the longer lever arm of the fused segments might accelerate degeneration [29, 61].

ASD is defined as radiographical changes at levels adjacent to a previous spinal fusion. ASD may manifest as cervical segment degeneration, such as disc degeneration, facet hypertrophy, segmental instability, et al.. In our simulation study, the average increase in stress values in the facet joint, the disc and the endplate of the Zero-P fusion model relative to the intact model were slightly lower than that of the standard PCC fusion model during flexion, extension, and lateral bending movement (Figs. 5, 6, 7 and 8). Increased stress might induce and aggravate degeneration of cervical facet joints and disc. The endplate also plays a key role in the development of disc degeneration, because most small molecule nutrient transport occurs via the endplate [68]. Load could regulate nutrient supply via the endplate. Disfunction of nutrition channel on cartilage endplate caused by abnormal stress intervention may be a contributory factor of cervical disc degeneration [69]. Therefore, larger stress in adjacent segments of the PCC model might lead to a higher incidence of ASD than in the Zero-P model.

Investigators have studied the risk factors for adjacent segment disease in spinal fusion. There are still disputes about the influence of plate-to-disc distance on ASD. Several clinical studies could not draw the conclusion that plate-to-disc distance affects the development of ASD [70], however, more and more studies have reported that plate-to-disc distance was independently associated with ASD, and the shortest plate is recommended to use to avoid intrusion of the plate close to the adjacent segments [27, 28, 71–73]. The Zero-P implant consists of a cage and an internal implant with a pair of locking screws. The greatest difference between the Zero-P and traditional PCC structures is that whether additional titanium alloys plate is attached to the anterior surface of the vertebral body. In the current computational simulation, at least 5 mm of plate-to-disc distance was adopted to simulate a standard ACDF surgical procedure. Our finite element study indicated that the standard PCC fusion model has a slightly larger stress transmitted to the adjacent segment than the Zero-P model. Compared to the traditional PCC model, the plate-to-disc distance in the Zero-P is longest. Therefore, the process of ASD might be influenced by the plate-to-disc distance. The biomechanical differences between the two models were small in the present study. This might be due to a single level fusion model was used in our study. The differences might be large in two or three level fusion situations.

### Limitations

There are some limitations in the present study. Although extra care was taken during model development and analysis, the finite element analysis has limitations, just like the cadaver studies and other published finite element studies. Caution should be taken when interpreting the results of the present study, because the intact FEM is based on a single scan of a normal man. The computational simulation aimed to provide the trend rather than the actual data. The comparison in the finite element analysis is not a statistical comparison. It is only a biomechanical trend analysis and comparison, similar to many finite element analysis researches. In our FEM, neck muscles were absence. The muscles mainly control the cervical range of motion. The absence of neck muscles may have some effect on the finite element biomechanical values, such as motion and stress. The fusion models were created and modified based on the healthy FEM. The cervical spine structures not to undergo surgery might be of different degenerative degree. Therefore, the conclusions of the present study should not be extrapolated to different genders or ages with varying degrees of degeneration. To improve the model and to study the effects of pre-exist degenerative changes in adjacent levels on the adjacent segment biomechanics after ACDF, mild, moderate and severe degeneration conditions should be simulated. Both the geometry and material properties should be changed. Height reduction, anterior osteophytes and endplate sclerosis should be incorporated into the geometry changes of different degeneration as the clinical basis. In addition to changing the geometry of the disc and the nucleus with altered mechanical properties, the FEM also includes changes in the permeability and porosity of various disc components and the reduction in nucleus water content. The effects of degenerative changes in adjacent levels on the biomechanical response of adjacent segment after ACDF should be studied further.

## Conclusion

In summary, this study showed that the ACDF, either with the Zero-P or with the standard PCC, could induce higher changes in the adjacent segment flexibility and in the disc, the endplate, and the facet stresses compared with the normal condition. Biomechanical factors related to the development of ASD progression are highest in the segment immediately above the fusion. The biomechanical features of the adjacent segment after fusion were most affected during extension. The finite element analysis indicated that the plate profile might affect the biomechanics of the adjacent-level after a single-level ACDF. The average increase of ROM and stress in the PCC model were slightly larger than that in the Zero-P model. The current findings may help explain the decreasing incidence of ASD complications in the patients using Zero-P compared with the patients using PCC.

## Data Availability

The datasets used and/or analyzed during the current study are available from the corresponding author on reasonable request.

## Abbreviations

ASD: Adjacent segment degeneration
ACDF: Anterior cervical decompression and fusion
FEM: Finite element model
ROM: Range of motion
Zero-P: Zero-profile spacer
PCC: Plate and cage construct
AF: Annulus fibrosus
NP: Nucleus pulposus
